# Challenges faced by Chinese community nurses when providing home-based hospice and palliative care: a descriptive qualitative study

**DOI:** 10.1186/s12904-022-00905-8

**Published:** 2022-02-01

**Authors:** Jinxin Zhang, Yingjuan Cao, Mingzhu Su, Joyce Cheng, Nengliang Yao

**Affiliations:** 1 School of Nursing and Rehabilitation, Cheeloo College of Medicine, Shandong University, Jinan, Shandong China; 2 Department of Nursing, Qilu Hospital of Shandong University, Jinan, Shandong, China; 3 Centre for Health Management and Policy Research, School of Public Health, Cheeloo College of Medicine, Shandong University, Shandong Jinan, China; 4 NHC Key Lab of Health Economics and Policy Research, Shandong University, Shandong, Jinan, China; 5 University of Virginia College of Arts and Sciences, Charlottesville, VA USA; 6 University of Virginia, School of Medicine, Charlottesville, VA USA

**Keywords:** Community nurse, Home-based hospice and palliative care, Challenge, Qualitative research

## Abstract

**Background:**

Hospital facilities in China are experiencing increased strain on existing systems and medical resources, necessitating the use of home-based hospice and palliative care (HBHPC). HBHPC primarily relies on community nurses and related medical personnel. Understanding the challenges that community nurses face when providing this form of care is urgently needed to optimize the design and delivery of HBHPC. Our study aimed to gain insight into community nurses’ challenges when providing HBHPC for patients.

**Methods:**

We performed a descriptive qualitative study using a phenomenology approach. Purposive sampling was used to recruit 13 nurses from two community health service centers in Jinan, Shandong Province, China. A thematic analysis was applied to identify themes from the transcribed data.

**Results:**

Three major themes emerged: 1) Community nurses’ inadequate self-preparation for providing HBHPC; 2) Patients and their families’ non-collaboration in HBHPC; 3) Community health service career disadvantages. Many negative experiences can be attributed to institutional barriers.

**Conclusion:**

Community nurses faced multifaceted challenges in home care settings. This study could provide a framework for guiding the improvement of interventional variables in the provision of HBHPC. Future research should involve developing effective methods of improving community nurses’ job motivation and community health service institutions’ incentive systems, as well as increasing advocacy around HBHPC.

## Introduction

Palliative care has been associated with relieving suffering and optimizing the quality of life among patients with poor disease prognoses and their families [1]. Existing studies suggest that most people prefer to receive care and to die at home, and a majority of patients do not change their opinion as their disease progresses [2]. The focus of hospice and palliative care should not only be on controlling physical symptoms but also on meeting the patient’s preference for their place of death [3, 4]. The Cochrane systematic review showed that home-based hospice and palliative care (HBHPC) increased the odds of dying at home [5]. Previous evidence has shown that patients who received HBHPC had lower symptom burdens, lower health care costs, and higher care satisfaction [6, 7]. Care at home fulfilled patients’ wishes to avoid excessive medical treatment and to achieve a dignified death [8, 9].

There are two main systems of providing HBHPC in China. One functions through hospitals, and the other relies on community health centers (CHCs) [10]. The former requires more hospital resources and time, and incurs higher patient costs that are not covered by health insurance [10]. Home-based hospice and palliative care offered by CHCs are a potential solution for easing current pressures on hospitals. HBHPC teams are composed of community nurses, general practitioners, and rehabilitation physicians who provide high-quality care for patients in residential communities with populations of around 30,000 people [11, 12]. A few healthcare professionals from each CHC are often selected to attend short training on hospice and palliative care [13].

In the past few years, some CHCs have begun to launch HBHPC services, which are often organized by nurses, general practitioners, rehabilitation therapists, and pharmacists. The municipal government has assisted communities in certifying eligibility for HBHPC recipients. Despite this progress, HBHPC programs have been difficult to implement due to insufficient legislation and policy, as well as a lack of financial, educational, and training resources [14]. Furthermore, most patients and their families have a low degree of knowledge and acceptance of hospice and palliative care, mostly due to the influence of traditional Chinese culture—discussing death remains taboo [15–17].

Home-based hospice and palliative care programs put higher demand on community nurses’ skills, because HBHPC moves specialized caring out of CHCs into the home environment [18]. The role of HBHPC community nurse is a relatively new phenomenon. The profession is not well-known, and neither are the challenges faced by these nurses. This lack of knowledge hinders efforts to evaluate how HBHPC can reach its full potential and support the national government’s public health goals. Understanding specific challenges experienced by community nurses when delivering patient and family-centered hospice and palliative care in home-based conditions may allow us to improve HBHPC quality and guide future healthcare delivery. Therefore, we aimed to explore the challenges faced by community nurses when providing HBHPC.

## Methods

### Aim

This study explored the challenges faced by community nurses who provide HBHPC.

### Study design

We used a descriptive phenomenology approach to explore the obstacles experienced by community nurses who provided HBHPC [19]. We conducted semi-structured, in-depth, face-to-face interviews to collect data between October 2018 and February 2019.

### Setting/participants

The study was conducted through two CHCs in Jinan, China. Shandong Province is the second-most populous province in China and its population faces a high disease burden [20]. The city of Jinan in Shandong Province is also home to a large elderly population with prominent features of aging and disability. Home-based patients present a substantial demand for hospice and palliative care [20].

We used purposive sampling for nurse selection. The eligibility criteria for study participation included: 1) registered nurses who have worked in the CHC for at least 1 year; 2) provided home-based care within 6 months of the study for patients who had a diagnosis of cancer, heart failure, or other life-limiting, progressive, or disabling diseases; 3) ability to speak Mandarin. Exclusion criteria were as follows: 1) nursing interns; 2) declined to participate in the interview. Each nurse was interviewed in a private room during their work time. No new themes emerged from participants’ experiences when the number of community nurses reached 14.

### Ethical approval

The Ethics Review Board of Shandong University’s School of Nursing and Rehabilitation approved this study (No. 2016-e-23). Participants gave fully informed consent by signing a consent form, and all documents were kept private and confidential. All audio-recorded interviews were reviewed by the transcriber and the principal investigator only, and each participant was identified by a specific code number rather than by name.

### Data collection

Two interviewers with qualitative research and interview skills conducted the entire interview process. Before data collection, the researcher visited the targeted CHCs. She gained the support of the CHCs’ leadership, established a trusting relationship with nurses, and learned about HBHPC.

The primary researcher then explained the purpose, methods, and significance of the study to potential participants; collected general demographic data; assured the interviewees of the anonymity and confidentiality of their information; and promised that the collected data would not affect participants’ work performance evaluations. After obtaining participants’ informed consent, interviews were conducted and recorded. Data collection involved semi-structured, personalized, face-to-face interviews with nurses performed in Mandarin Chinese by the primary researcher, who had background experience in nursing, allowing for better understanding of the participants’ perspectives. The other researcher took notes during the interview, which were used to provide contextual information concerning the interview for future reference.

The interview outline was developed through literature review and consulting professionals, and our research group discussed the question outline in advance. The interview started with two open-ended questions, and then follow-up questions were asked to clarify participants’ viewpoints.

The interview’s two main questions were as follows:

1. What are your experiences of providing home-based hospice and palliative care?
2. Based on your experiences, what are the challenges, difficulties, confusions, and conflicts you have faced when providing home-based hospice and palliative care?

### Data analysis

The researcher used a thematic analysis approach to analyze the interview data [21]. Within 24 h after the end of each interview, the researcher listened to the recording, referred to the on-site notes, and transcribed the recording verbatim. The “conformity method” was adopted for data analysis [21]. The researcher carefully read the data; deliberated on the recording and text content; coded, classified, reasoned, and analyzed the recurring content; and finally extracted the theme. She consulted another researcher about uncertainties regarding themes, and disagreements were discussed until consensus was reached among the research team. Finally, initial themes were communicated with participants in one-on-one face-to-face meetings [21]. Our reporting adhered to the consolidated criteria for reporting qualitative research (COREQ) guidelines [22].

## Results

### Socio-demographic characteristics

A total of 13 nurses from the two CHCs participated in our research. The nurses’ mean age was 37.2 years old, and their ages ranged from 25 to 53 years old. Their experience in providing medical care before these interviews ranged from 3 to 29 years, and their HBHPC experiences ranged from 3 to 12 months. Three community nurses had been offered bianzhi (lifelong tenure) and 10 were not tenured [23]. Table 1 summarizes the characteristics of the participants.

**Table 1:** Participant characteristics (*N* = 13)

Characteristics of participants	Descriptions	N
Age range	25–29	3
	30–39	5
	40–49	3
	50–53	2
Gender	Female	13
	Male	0
Highest educational level	Junior college degree holder	7
	Bachelor degree holder	5
	Master degree holder	1
Bianzhi^a^	Yes	4
	No	9
Length of nursing service (years)	3–10	4
	11–20	5
	21–30	4
Length of home-based hospice and palliative care service (months)	< 6	2
	≥6	11

^a^Bianzhi is similar to the concept of lifelong tenure guaranteed by government funding

### Analysis results

Analysis of the data using a phenomenological research method and thematic analysis method revealed the following three themes: 1) Community nurses’ inadequate self-preparation for providing HBHPC; 2) Patients and their families’ non-collaboration in HBHPC; 3) Community health service career disadvantages (Table 2).

**Table 2:** Summary of all primary and sub-themes extracted from the interviews

Main themes	Subthemes
**Community nurses’ inadequate self-preparation for providing HBHPC**	(i) Community nurses’ low job motivation toward providing HBHPC (ii) Community nurses’ inadequate professional ability to provide HBHPC
**Patients and their families’ non-cooperation in HBHPC**	(i) Patients and their families’ behaviors of poor adherence to HBHPC (ii) Patients and their families’ unaccepting attitudes toward HBHPC
**Community health service career disadvantages**	(i) Lack of career development opportunities (ii) Inadequate benefits

*HBHPC* home-based hospice and palliative care

### Community nurses’ inadequate self-preparation for providing HBHPC

#### Community nurses’ low job motivation

While nurses considered HBHPC critical for patients with incurable diseases, it was recognized as a new form of nursing and a practice that nurses did not feel they were adequately prepared for. Most of them said that they only passively accepted the work tasks assigned by superiors.

*“I just passively complete my work [home-based hospice and palliative care]. It is just a job for me. I am only here to make money.” (N3)*

Some nurses described that, compared with nurses in hospitals, home care nurses had lower social status. They were rejected by patients and their family members due to a societal emphasis on medical treatment over nursing care, which made them less motivated to work.

*“When I went to visit patients in a white coat, they would think that I was a doctor and they would welcome me, but when I went in a nurse uniform, I felt discriminated against.” (N6)*

### Community nurses’ inadequate professional ability to provide HBHPC

Community health centers offered courses in nursing theory regularly, but the nurses considered these courses superficial. Many nurses identified particular challenges in providing HBHPC, such as being unable to deliver highly individualized patient and family-centered care. It was difficult for nurses to identify their patients’ psychological challenges at different phases of their illness (e.g., soon after being diagnosed with an incurable disease, when living with the terminal illness, and at or near the terminal stage). Thus, it was difficult for them to take the initiative to prevent and relieve patients’ anxiety, depression, and distress. Community nurses were supposed to address families’ cultural preferences and requirements in their practice, but there were few opportunities for assessing and understanding family relationships. Nurses could only build on their prior personal experiences working in CHCs, as they lacked advanced training.

*“It is hard for me to provide family-centered care for home-bound patients with more complex needs. For example, some family caregivers are rude to patients and do not practice good domestic hygiene… We did not know much about the patient’s religious beliefs and cultural preferences.” (N3)*

*“It is undeniable that I lack the experience and knowledge of psychological care. In addition, I worry that patients and caregivers may think I am weird if I talk about spiritual care (since nurses don’t do that in China).” (N12)*

### Patients and their families’ non-collaboration in HBHPC

#### Patients and their families’ behavior of poor adherence to HBHPC

The participants stated that patients’ actual health behaviors might not conform to their medical team’s recommendations. Sometimes, patients and their families even refused to accept treatment because they didn’t understand what was wrong with them or how the medical staff could help them.

*“After a professional assessment of the patient, we wanted to give him some traditional Chinese medicine treatment for his persistent lower extremity edema and constipation, but he did not think he needed it.” (N8)*

*“It was time to replace the nasogastric tube, but family members were unwilling to let us perform this operation. They asked me to come again next Wednesday.” (N5)*

Participants found that some patients’ families were not heavily involved in basic illness management and could not provide adequate affection or support. Relatives might be unable or unwilling to care for their home-bound elderly family members for a long time, and family members may rationalize their behavior by blaming others. However, nurses felt like these issues were private affairs of the patient’s family, and it was inconvenient to mediate between patients and families regarding these conflicts. This tension affected nurses’ willingness to continue visiting these patients.

*“It is difficult for clinicians to intervene in family conflicts. Adult children normally take turns to care for the homebound parent, but some may be reluctant to do that. They are also not willing to pay the medical and living expenses of their elderly parent for an extended time. They use the excuse that they have a demanding, stressful job, so they do not want to take their turn to care for their parents.” (N1)*

*“This home-bound patient has always been taken care of by his youngest son. I asked the patient’s other son to buy some cotton swabs to help the patient moisturize his lips and mouth, but he was playing game on a smartphone and said he didn't have time.” (N2)*

#### Patient and their families’ unaccepting attitudes toward HBHPC

Community nurses believed that the nurse-family relationship mainly depended on the patient and their family’s attitude towards palliative care. Although the nurse’s place in the home setting has the potential to become well-established through multiple visits, nurses were often rejected by patients and their families, especially in their first visits to patients’ homes. Nurses felt like they were disturbing the patients and their families.

*“When I knocked, they refused to open the door because they thought I was an unsolicited salesperson, I do not understand them, and they do not understand me… they did not trust us.” (N5)*

*“Before, I introduced myself at the door. The nurse-patient relationship was full of uncertainty at the initial visit; I didn’t know if patients and their families would let me in, or believe me, I just don’t know.” (N9)*

### Community health service career disadvantages

#### Lack of career development opportunities

Participants desired the same career development and training opportunities offered to public hospital nurses. Compared with public hospital nurses, who are usually required to have a bachelor’s or master’s degree, community nurses only need to have a junior college degree. Community nurses are rarely given time to obtain an advanced degree or achieve higher academic qualifications. Community nurses were very rarely offered bianzhi. Some nurses stated that there are only 1 or 2 new bianzhi per year within one CHC. Senior nurses who have been employed the longest, typically older nurses, are generally given priority, so there are limitations in career development and promotion for young community nurses.

*“[Compared to hospital nurses,] we [community nurses] have fewer opportunities to receive continuing education and obtain a higher professional nursing title. I just want to finish my job.” (N10)*

*“They [community nurses] thought of themselves as workers who were not offered bianzhi (lifelong tenure), so they lacked a sense of belonging. It was easy for them to deliver task-centered rather than patient-centered care in the process of providing home-based care. It affected the quality of home-based hospice and palliative care.” (N11)*

### Inadequate benefits

Many nurses had stated that they were not satisfied with their current salaries and the additional subsidies they received for providing HBHPC. More importantly, CHCs had not provided them with the necessary transportation and communication equipment needed to deliver home care. All nurses had to buy these materials at their own expense. Fringe benefits, such as transport and communication allowances, were the most frequently mentioned factor negatively affecting nurses’ motivation and intention to provide HBHPC.

*“HBHPC is not a task to be taken lightly and will take a tremendous amount of time and effort to achieve, but I received minimal fringe benefits from the community health service center. We don’t charge the family for psychological care. It’s hard not to lose my motivation.” (N4)*

*“We have to use our own electric bikes, mobile phones, and data plans. These were all paid for by ourselves… If I use my personal phone number to contact them, I worry the patient and family would call me too often.” (N7)*

## Discussion

This research used a descriptive qualitative approach to explore the challenges faced by community nurses when providing HBHPC in the prefecture-level city of Jinan in China’s Shandong Province. This study found that major challenges included nurses’ inadequate self-preparation to provide HBHPC, patients and their families’ non-cooperation in HBHPC, and community health service career drawbacks. Some of the negative experiences may be related to institutional barriers. Moreover, according to participants’ responses, we noticed that external problems that arose from the patients and institutions involved in HBHPC exacerbated the internal challenges, such as low motivation, faced by the nurses themselves.

The first central theme was related to nurses’ personal issues, and was subdivided into low job motivation and professional inability to provide HBHPC. The results were similar to those of published studies [24–26]—a lack of HBHPC knowledge and skills among health specialists was a common challenge when delivering primary care. HBHPC is a new form of nursing practice that nurses felt they were not sufficiently prepared for. According to organization theory [27, 28], new approaches to HBHPC likely entailed organizational changes. These variations subsequently created feelings of insecurity and confusion among the nurses involved related to care coordination, multidisciplinary collaboration, and expectations about their own and other specialists’ roles [27, 28]. Previous studies have shown that educating nurses about the benefits of HBHPC could increase their understanding and motivation to serve [29]. Knowledge of and belief in the benefits of HBHPC are major predictors and explanatory variables that affect nurses’ willingness to provide HBHPC [29]. Improved HBHPC teaching materials, provision of ongoing in-service training opportunities, and regular supervision could improve community nurses’ confidence when providing HBHPC [29–31].

Community nurses faced difficulties related to some instances of non-cooperation from patients and their families. Home care nurses are entering a family-centered care system. In HBHPC, nurses do not solely tend to patients—families are often significant participants in patients’ care, and family involvement should be encouraged [18]. One critical strategy for providing patient and family-centered care is sharing decision-making [32, 33]. The patient or their families and nurses should share essential information, discuss risks versus benefits of various nursing options, and express their preferences [32, 33]. Effective patient-nurse communication that permits collaborative patient and family-centered care improves adherence to medical advice, leading to better palliative care outcomes [32, 33].

Patients and their families’ potential biases exacerbated nurses’ lack of motivation and confidence when providing HBHPC. Public biases tend to favor physicians over nurses, as many believe that nursing simply entails executing doctors’ orders, and lacks credibility [34, 35]. Besides enhancing nurses’ abilities of HBHPC [36, 37], HBHPC requires that providers have service-oriented motivations [38], which include altruism, humanitarian respect, seeking new experiences, wanting to help others, and personal growth [39]. Altruism and humanitarianism often are influential factors that drive one to volunteer to make a difference in others’ lives [40]. However, regardless of whether nurses embodied these characteristics, many felt unmotivated to provide HBHPC, as the increased stress and effort required to deliver psychological care and family-centered care to patients were not reflected in their salaries.

Palliative care is typically a heavily multidisciplinary practice; however, home-based services only involved nurses, general practitioners, rehabilitation specialists, and pharmacists. Effective HBHPC requires the support of additional professionals, such as hospice and palliative care volunteers and social workers, who can assist with chores and tasks in the home setting [41]. Hospice palliative care volunteers can collaborate with nurses [41–43]. They positively influence quality of care for patients and their families by reducing stress, and by offering practical assistance, emotional support, and companionship [41–43]. Integrating these volunteers into the home setting would increase the number of individuals providing perspectives on care interventions, leading to more holistic, effective care [41–43], especially when it is inconvenient for nurses to participate in the patient’s family affairs.

Many community nurses mentioned that providing HBHPC increased their workload compared to their previous jobs with CHCs. They highly valued jobs with bianzhi, as bianzhi for them meant being considered an employee of the state administration, which is viewed as a more stable career. Participants were dissatisfied with their current career development opportunities and benefits; this feeling eventually decreased their motivation and willingness to provide HBHPC. The pay-return imbalance model suggests that providing more energy and effort than received in return produces a sense of psychological imbalance, which leads to negative emotions and sustained stress responses [44]. Increased effort-reward imbalance is an incremental predictor of burnout [45]. The public government should increase the financial support provided to CHCs and improve performance assessment indicators. Additionally, nurses’ job performance relied on incentive mechanisms to a certain extent. Incentive management is an essential part of the field, and incentives create benefits including retention of employees and higher motivation levels from nurses. Effective incentives can ensure the continued vitality of organizational development and promote organizational goals. Community nurses should receive external rewards (wage income, fringe benefits, etc.) and internal rewards (encouragements, praise, training, evaluation, etc.) commensurate with their work responsibilities.

The World Health Organization (WHO) suggests that community nurses and other CHWs can be guided to provide palliative care education to homebound patients and their families [46]. Hospice and palliative care courses should be part of continuing education available to community health workers (CHWs,) and the availability and accessibility of HBHPC education and training must be improved [46]. Chinese health authorities can focus on developing a public health care system involving nurse-led palliative home care teams with supervision by hospice-based palliative care units thereby improving the efficiency and effectiveness of education on palliative care. The Chinese government can design a policy system that considers HBHPC part of community-oriented primary health care, based on the Essential Package of palliative care for Primary Health Care (EP PHC,) to eliminate institutional barriers to the development and availability of HBHPC [47].

### Strengths and limitations

In our study, purposeful sampling was used to ensure inclusion of community nurses of differing ages, lengths of nursing service, and varying levels of education. Although all participants were female, this does somewhat reflect the typical gender composition of nurses with HBHPC experiences. Our study produced a wealth of data that deepened our understanding of the HBHPC experience and challenges nurses faced when providing this form of care. Another limitation is that all participants were recruited from two CHCs in Jinan. Thus, participants’ challenges when providing HBHPC might not represent community nurses’ experiences in other cities. Exploration of the experiences and challenges of community nurses in different, more diverse settings is needed.

## Conclusion

Our study generated three core themes related to community nurses’ challenges when providing HBHPC. Our research suggested that better self-preparation and increased organizational support are needed for nurses providing this form of specialized care. This study could provide a framework reference to guide the improvement of interventional variables in HBHPC. Future research should focus on improving nurses’ motivation to enhance their enthusiasm for their work, and on increasing advocacy around HBHPC.

## Data Availability

The datasets used and/or analyzed during the current study are available from the corresponding author on reasonable request.

## Abbreviations

HBHPC: Home-Based Hospice and Palliative Care
CHCs: Community Health Centers
