# Correction: Challenges faced by Chinese community nurses when providing home-based hospice and palliative care: a descriptive qualitative study

**DOI:** 10.1186/s12904-022-00995-4

**Published:** 2022-06-06

**Authors:** Jinxin Zhang, Yingjuan Cao, Mingzhu Su, Joyce Cheng, Nengliang Yao

**Affiliations:** 1 School of Nursing and Rehabilitation, Cheeloo College of Medicine, Shandong University, Jinan, Shandong China; 2 Department of Nursing, Qilu Hospital of Shandong University, Jinan, Shandong China; 3 Centre for Health Management and Policy Research, School of Public Health, Cheeloo College of Medicine, Shandong University, Jinan, Shandong China; 4 NHC Key Lab of Health Economics and Policy Research, Shandong University, Jinan, Shandong China; 5 University of Virginia College of Arts and Sciences, Charlottesville, VA USA; 6 Johns Hopkins University, School of Medicine, Baltimore, MD USA

**Correction: BMC Palliative Care 21, 1 (2022)**

**https://doi.org/10.1186/s12904-022-00905-8**

Following the publication of the original article [1], the co-author reported that her affiliation is incorrect in the published article. “University of Virginia, School of Medicine, Charlottesville, VA, USA.” should be “Johns Hopkins University, School of Medicine, Baltimore, MD, USA.” for the fourth author.

The affiliation has been corrected above and the original article has been updated.
